# Real-world outcomes and corticosteroid sparing with mepolizumab for EGPA or HES

**DOI:** 10.1016/j.jacig.2026.100695

**Published:** 2026-03-27

**Authors:** Michael E. Wechsler, Anna Kovalszki, Brian Stone, Jared Silver, Lior Seluk, Jeremiah Hwee, Lynn Huynh, Wilson da Costa Junior, Shuang Wang, Mei Sheng Duh, William McCann, Amy G. Edgecomb

**Affiliations:** a Division of Pulmonary, Critical Care and Sleep Medicine, National Jewish Health, Denver, Colo; b Division of Allergy and Clinical Immunology, Department of Internal Medicine, University of Michigan, Ann Arbor, Mich; c Allergy Partners, Asheville, NC; d US Medical Affairs–Respiratory, GSK, Durham, NC; e Global Epidemiology, GSK, Mississauga, Ontario, Canada; f Analysis Group, Inc, Boston, Mass; g US Real-World Evidence & Health Outcomes Research, Anti-Infectives and Respiratory, GSK, Philadelphia, Pa

**Keywords:** Eosinophilic granulomatosis with polyangiitis, hypereosinophilic syndrome, mepolizumab, oral corticosteroids, clinical outcomes, end-organ manifestations, relapse, remission, steroid sparing, private practice

## Abstract

**Background:**

Eosinophilic granulomatosis with polyangiitis (EGPA) and hypereosinophilic syndrome (HES) are rare systemic inflammatory diseases with overlapping features. Mepolizumab, a humanized mAb targeting IL-5, has been approved for treatment of both conditions. However, real-world data on its clinical benefit in private practice settings remain limited.

**Objective:**

We sought to evaluate oral corticosteroid (OCS) use, clinical outcomes, and end-organ manifestations post-mepolizumab initiation in private practice settings using electronic medical records.

**Methods:**

This retrospective pre-post cohort study used data from the US-based Allergy Partners network electronic medical records (January 1, 2007, to August 17, 2023) to select patients with EGPA or HES who initiated mepolizumab (index date). Participants were 18 years and older (EGPA) or 12 years and older (HES) with 3 or more months of clinical activity pre- and postindex. Outcomes included OCS use and dose, clinical outcomes (response and remission), and end-organ damage manifestations.

**Results:**

Overall, 44 patients with EGPA and 16 patients with HES had significant reductions in OCS use and dose (*P* = .014 for trend over each 6-month period), with improvements in end-organ manifestations over a treatment period up to 2 years. Among patients with EGPA, clinical response, disease control, and remission rates increased significantly. In addition, 86.4% of patients with EGPA experienced an improvement in at least 1 of the outcome measures of OCS use, end-organ manifestations, or blood eosinophil count. Among patients with HES, end-organ manifestations were more common preindex (87.5%) than postindex (56.3%).

**Conclusions:**

These findings highlight the long-term OCS-sparing effects of mepolizumab and suggest additional clinical benefits for patients with EGPA and HES in a private practice setting.

Eosinophilic granulomatosis with polyangiitis (EGPA) and hypereosinophilic syndrome (HES) are rare systemic inflammatory diseases with substantial overlap in clinical presentation.^1^ Although the clinical features of each disease are heterogeneous, both are characterized by elevated eosinophil counts and eosinophil tissue infiltration.^1^, ^2^, ^3^ Furthermore, both diseases are associated with a substantial burden of disease, with patients experiencing symptoms across a number of organ systems, which can result in end-organ damage.^4^

Most studies of patients with EGPA and HES have focused primarily on characterizing patients within the setting of specialist referral or tertiary care centers, although a considerable number of patients with HES and EGPA are also treated in private practice settings.^5^, ^6^, ^7^ Treatment of EGPA or HES relies heavily on oral corticosteroids (OCSs) as first-line therapy. However, these are associated with numerous adverse effects, which add to the patient burden.^6^, ^7^, ^8^, ^9^ Consequently, OCSs are often used in combination with other immunosuppressive agents and biologics targeted at eosinophil regulation and maintenance, with the aim of reducing OCS toxicity.^2^^,^^4^^,^^10^^,^^11^ Despite the emergence of targeted therapies, recent observational data confirmed high OCS use in HES and EGPA.^5^^,^^7^^,^^12^

Mepolizumab is a first-in-class humanized mAb specifically targeting IL-5 and is approved for the treatment of EGPA, HES, severe asthma with an eosinophilic phenotype, and chronic rhinosinusitis with nasal polyps in multiple regions worldwide.^13^, ^14^, ^15^ The benefits of IL-5 inhibition with mepolizumab for patients with EGPA or HES have been established in phase 3 placebo-controlled trials and their open-label extensions.^16^, ^17^, ^18^, ^19^ In both EGPA and HES, IL-5 inhibition with mepolizumab significantly reduced blood eosinophil counts, increased remission (EGPA), and reduced relapse rates (EGPA) or flares (HES), while also reducing the need for OCSs, compared with placebo. However, real-world studies evaluating the impact and clinical benefits of mepolizumab are limited, particularly in private practice settings.^1^^,^^6^^,^^19^^,^^20^

This study aimed to evaluate clinical outcomes (clinical response, control status, remission, and relapse rates), changes in OCS use, and end-organ manifestations following initiation of mepolizumab in patients with EGPA or HES using electronic medical records (EMRs) from the Allergy Partners network (the largest network of private allergy practices in the United States) to report meaningful evidence from real-world experience.

## Methods

This was a retrospective pre-post cohort study (GSK ID: 218960) of patients treated in the Allergy Partners network with a confirmed diagnosis of EGPA or HES who received mepolizumab (see Fig E1 in this article’s Online Repository at www.jaci-global.org). Patients were identified from 1.7 million patients in the Allergy Partners EMRs from January 1, 2007, to August 17, 2023. The index date was defined as the first mepolizumab initiation (100 or 300 mg subcutaneously) during the study period. The preindex period was defined as the 6-month period before the index date, and the postindex period was defined as the time from the index date until death, discontinuation of mepolizumab, receipt of another biologic, or end of data or as the 6-month period after the index date, whichever came first. Data were collated from structured EMRs and unstructured clinical notes from the Allergy Partners electronic health records database.

### Participants

Patients with a confirmed diagnosis of EGPA were 18 years and older in the index year, and those with a confirmed diagnosis of HES were 12 years and older in the index year. Patients in both disease cohorts initiated mepolizumab after EGPA or HES diagnosis and within the study period, had 3 months or more of clinical activity before the index date (defined as the patient having ≥1 clinical encounter >3 months before the index date), and had 3 months or more of clinical activity after the index date (defined as the patient having ≥1 clinical encounter >3 months after the index date), unless the patient died. A clinical encounter was a documented patient visit with a provider in the Allergy Partners network and/or receipt or administration of medications, as documented in the EMRs.

Diagnosis of EGPA was confirmed in 2 steps—first, identification of *International Classification of Diseases, Ninth/Tenth Revision* diagnosis code or designation of “EGPA” or “Churg−Strauss” in the EMR problem description field, followed by a confirmation based on 2 or more additional features of EGPA in the clinical notes (adapted from the MIRRA trial inclusion criteria).^2^^,^^17^ These comprise biopsy showing histopathologic evidence of eosinophilic vasculitis, perivascular eosinophilic infiltration, or eosinophil-rich granulomatous inflammation; asthma diagnosis; vasculitis; mono- or polyneuropathy (motor deficit or nerve conduction abnormality); pulmonary infiltrates (nonfixed); sinonasal abnormality; cardiomyopathy; glomerulonephritis; alveolar hemorrhage; palpable purpura; or a positive antineutrophil cytoplasmic antibody screen.

HES diagnosis was identified by the presence of *International Classification of Diseases, Tenth Revision* diagnosis codes or a designation of “hypereosinophilic” or “HES” in the EMR problem description field followed by a confirmatory step for clinical features on the basis of blood eosinophilia (eosinophil count ≥1500 cells/μL) and no discernable secondary cause (eg, allergic diseases, drug hypersensitivity, parasitic helminth infections, HIV infection, or nonhematologic malignancies).

### Outcomes

Outcomes were compared for the pre- versus postindex periods for patients with EGPA and HES separately. The primary outcome was the proportion of patients prescribed OCSs treated within the Allergy Partners EMR database in the pre- and postindex periods. Secondary outcomes included mean daily doses of OCSs (calculated as prednisone-equivalent doses) in the pre- and postindex periods and the incidence rate of OCS prescriptions.

Clinical outcomes in terms of response, control status and/or remission, including stringent remission (EGPA only), and relapse among patients with EGPA or flare episodes among patients with HES over the 2 years preindex and 2 years postindex were also assessed on the basis of clinical notes. Complete response was defined as physician-reported improved or controlled symptoms and normal blood eosinophil count (≤500 cells/μL) recorded within 30 days of each other. Control status was determined by physician assessment of first controlled status or controlled status after worsened, unchanged, or active symptoms. Patients who were categorized as having a response experienced symptom improvement, whereas those achieving control status achieved controlled symptoms. Remission was defined as prednisone-equivalent OCS dose less than or equal to 4 mg/d among those who had control status (adapted from the MIRRA trial primary remission definition)^17^; additional remission definitions with more restrictive criteria were applied as a sensitivity analysis (either no OCS use or no OCS use without relapse after achieving remission). Stringent remission was defined as achieving controlled status, no OCS use, and having no relapse within 2 years of mepolizumab initiation. Relapse among patients with EGPA was defined as physician-reported worsening of active symptoms with any previous assessment of controlled status and any of the following (contingent on data availability): any increase in OCS dose, an increase/switch in immunosuppressants, or hospitalization for EGPA. Flares among patients with HES were defined as the worsening of HES-related clinical symptoms and blood eosinophil count requiring therapy escalation (such as additional therapy for HES).

Although full Birmingham Vasculitis Activity Scores (BVASs) could not be generated because of insufficient data, individual components were evaluated. Among patients with EGPA who achieved remission (achieved control status and receiving OCS ≤4 mg/d), BVAS-related clinical manifestations were assessed by describing the proportion of patients with individual manifestations within 4 weeks before the date of remission.

End-organ damage manifestations (cardiac, pulmonary, renal, neurologic, skin, and gastrointestinal) were also assessed pre- and postindex on the basis of available data from diagnostic tests/biopsies and were classified by postindex status as improved, unchanged, worsened, or unknown. Neurologic, cardiac, gastrointestinal, pulmonary, and skin-manifestation changes were confirmed through imaging, laboratory results, biopsies, and clinical notes. Further outcomes comprised proportion of patients prescribed immunosuppressants, symptom occurrence and rate, in addition to pulmonary and symptom scores in patients with comorbid asthma (asthma control test symptom and daily functioning score and spirometry parameters).

### Data sources and statistical analysis

Data were compiled from the proprietary clinical database of the Allergy Partners EMR system, the largest single-specialty practice in allergy, asthma, and immunology in the United States. The database contains structured data and extensive unstructured data included in provider notes and clinical reports. OCS and immunosuppressant use, laboratory assessments, and end-organ manifestations (excluding evolvement of manifestations) were evaluated using both structured and unstructured data. Clinical outcomes and evolvement of end-organ manifestations were evaluated using unstructured data, and symptoms were evaluated using structured data.

Data were analyzed for 4 cohorts: patients with EGPA receiving mepolizumab at either dose (100 or 300 mg), patients with HES receiving mepolizumab (either dose), and for the 2 subgroups of patients who received mepolizumab doses of 300 mg in the EGPA and HES cohorts. Outcomes were compared pre- versus post-mepolizumab initiation. All analyses were conducted using SAS version 9.4 (SAS Institute, Inc, Cary, NC).^21^

Patient demographic characteristics, disease characteristics and comorbidities, and laboratory tests during the preindex period were determined using descriptive statistics. Frequency and proportion of patients receiving OCSs pre- versus post-mepolizumab initiation were compared using the McNemar test. The Wilcoxon signed rank test was performed to compare OCS mean daily dose, immunosuppressant prescriptions, asthma control test scores, and spirometry parameters pre- versus post-mepolizumab. Clinical outcomes (response, control status, and remission) pre- and postindex were compared using the McNemar test. In addition, descriptive statistics were used to report end-organ damage manifestations pre- and postindex. Trend analyses using the Mann-Kendall Trend test were performed to assess the number of patients using OCSs as well as the OCS daily dose over each 6-month time period from 6 months pre-mepolizumab initiation until 24 months after initiation or end of follow-up.

### Ethics and study conduct

The study protocol was approved by a central institutional review board (IRB) before data collection (IRB ID: 2023-0360). The study was conducted entirely using retrospective medical records, and primary data collection was not necessary; exemption was obtained from Pearl IRB (Indianapolis, Indiana, USA). Informed consent was not required, because the data were fully deidentified in compliance with the Health Insurance Portability and Accountability Act of 1996. The physicians who performed chart review removed all identifying data before submitting it for analysis and the minimum retention time met the strictest standard applicable for the study.

## Results

### Patient population

Overall, 44 eligible patients with EGPA and 16 with HES were identified (see Figs E2 and E3 in this article’s Online Repository at www.jaci-global.org). For the EGPA cohort, the mean age at index was 51.8 ± 15.2 years, with a slightly higher proportion of females (Table I). In the HES cohort, the mean age was 54.6 ± 19.3 years, with an even split between males and females, of whom 10 (62.5%) had idiopathic disease. The median (Q1, Q3) baseline blood eosinophil count was 745.0 (383.0, 1491.5) cells/μL in the EGPA cohort and 1205.0 (264.0, 1655.6) cells/μL in the HES cohort. More than 80% of the EGPA cohort had comorbid conditions of the respiratory system at index, with 43 of 44 patients having a documented diagnosis of asthma at any time pre- or postindex. In the HES cohort, 81.3% had respiratory system conditions, comprising predominantly asthma and rhinitis. In addition, 3 patients had an overlap of EGPA and HES diagnoses and were included in both cohorts. During the preindex period, 39 (88.6%) patients in the EGPA cohort and 14 (87.5%) patients in the HES cohort received OCSs (Table I).

**Table I:** Patient demographic and clinical characteristics

Patient population	EGPA cohort (N = 44)	HES cohort (N = 16)
*Demographic characteristics at index*		
Age (y), mean ± SD	51.8 ± 15.2	54.6 ± 19.3
Sex: female, n (%)	26 (59.1)	8 (50.0)
Race, n (%)		
White	31 (70.5)	9 (56.3)
American Indian	2 (4.5)	2 (12.5)
Black or African American	2 (4.5)	2 (12.5)
Asian	2 (4.5)	0 (0.0)
Unknown	7 (15.9)	3 (18.8)
Ethnicity, n (%)		
Non-Hispanic	34 (77.3)	14 (87.5)
Hispanic	7 (15.9)	2 (12.5)
Unknown	3 (6.8)	0 (0.0)
Payer type, n (%)		
Commercial	28 (63.6)	6 (37.5)
Medicare	14 (31.8)	8 (50.0)
Self-insured	1 (2.3)	0 (0.0)
Medicaid	1 (2.3)	1 (6.3)
Other	0 (0.0)	1 (6.3)
*Clinical characteristics at index*		
Diagnosis of EGPA/HES within Allergy Partners, n (%)		
Yes	20 (45.5)	7 (43.8)
No	24 (54.5)	9 (56.3)
EGPA disease phase^22^ (at most recent date before/on initiation), n (%)		
Vasculitic∗	18 (40.9)	—
Eosinophilic†	18 (40.9)	—
Prodromal‡	6 (13.6)	—
Unknown	2 (4.5)	—
HES disease subtype,§ n (%)		
Idiopathic	—	10 (62.5)
Lymphocytic	—	3 (18.8)
Myeloid	—	1 (6.3)
Unknown	—	2 (12.5)
Blood eosinophil count‖		
Patients with ≥1 assessment, n (%)	40 (90.9)	16 (100.0)
Baseline blood eosinophil count (cells/μL), median (Q1, Q3)	745.0 (383.0, 1491.5)	1205.0 (264.0, 1655.6)
Time from the closest assessment to index date (mo), median (Q1, Q3)	4.1 (1.3, 12.4)	5.0 (0.8, 7.3)
ANCA status,† n (%)		
≥1 assessment	23 (52.3)	7 (43.8)
Positive result	5 (21.7)	0 (0.0)
MPO	2 (40.0)	—
PR3	2 (40.0)	—
Unknown	1 (20.0)	—
Negative result	18 (78.3)	7 (100.0)
Comorbidities, n (%)		
Respiratory system	36 (81.8)	13 (81.3)
Gastrointestinal system	14 (31.8)	12 (75.0)
Circulatory system	5 (11.4)	3 (18.8)
Nervous system	2 (4.5)	3 (18.8)
Anxiety and depression	—	1 (6.3)
Infectious diseases	2 (4.5)	2 (12.5)
Patients with EGPA/HES overlap, n (%)	3 (6.8)	3 (18.8)
Patients with OCS use,† n (%)	39 (88.6)	14 (87.5)

*ANCA*, Antineutrophil cytoplasmic antibody; *FISH*, fluorescence *in situ* hybridization; *MPO*, myeloperoxidase; *PR3*, proteinase 3.

∗ Constitutional symptoms such as fever, weight loss, fatigue, and vasculitis of small- to medium-sized vessels.

† Eosinophilia in the peripheral blood and tissues without proven vasculitis.

‡ Allergic rhinitis, recurrent sinusitis, and nasal polyposis.

§ On the basis of genetic data available on FISH panel.

‖ Any time before index.

Mean duration of mepolizumab treatment postindex was 2.4 ± 1.7 years for patients with EGPA and 1.7 ± 1.8 years for patients with HES (Table II). During the postindex period, 30 (68.2%) patients in the EGPA cohort and 10 (62.5%) patients in the HES cohort received mepolizumab doses of 300 mg throughout, with 4 (9.1%) and 2 (12.5%) patients, respectively, increasing from 100 to 300 mg (Table II).

**Table II:** Characteristics of mepolizumab use

Patient population	EGPA cohort (N = 44)	HES cohort (N = 16)
Time from first clinical activity to mepolizumab initiation (mo), median (Q1, Q3)	29.9 (9.0, 64.0)	19.4 (15.4, 28.9)
Time from mepolizumab initiation to last clinical activity (mo), median (Q1, Q3)	31.3 (17.5, 51.5)	25.1 (16.8, 47.5)
Mepolizumab dosage, n (%)		
Always 300 mg	30 (68.2)	10 (62.5)
Always 100 mg	10 (22.7)	4 (25.0)
Increased from 100 to 300 mg	4 (9.1)	2 (12.5)
Duration of mepolizumab treatment postindex (y),∗ mean ± SD	2.4 ± 1.7	1.7 ± 1.8
Discontinued during postindex follow-up	10 (22.7)	6 (37.5)

∗ Duration to earliest of death, mepolizumab discontinuation, receipt of another biologic, end of clinical activity, or end of data.

### OCS treatment patterns

**Fig 1:**
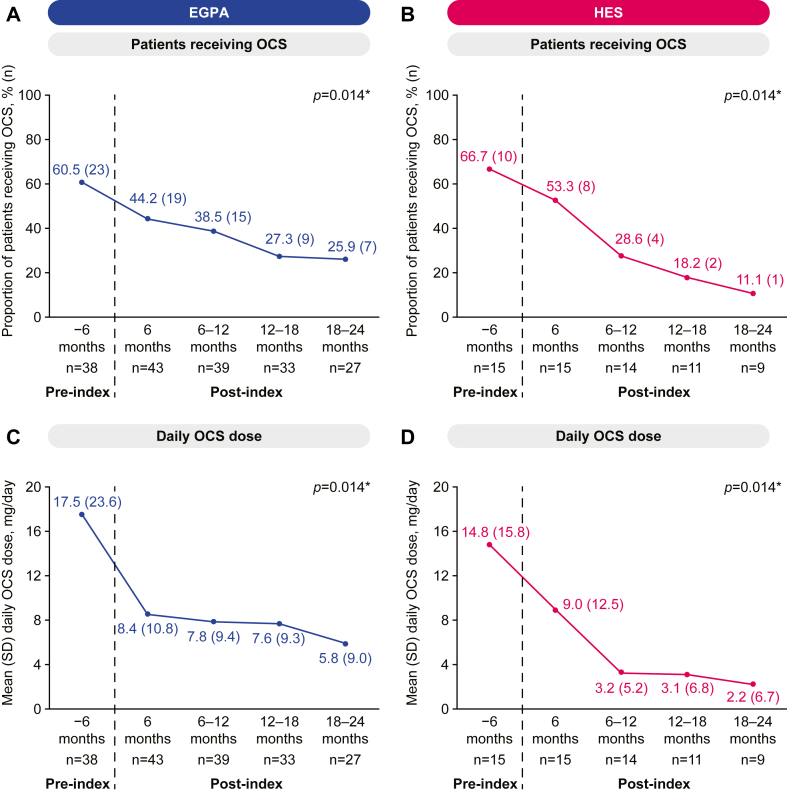
**A-D,** OCS use among patients with EGPA (Fig 1, *A* and *C*) or HES (Fig 1, *B* and *D*) from 6-month premepolizumab initiation up to 24-month post-mepolizumab initiation: proportion of patients receiving OCSs (Fig 1, *A* and *B*) and mean OCS daily dose (Fig 1, *C* and *D*). ∗*P* < .05 for trend over each 6-month period.

In both the EGPA and HES cohorts, the proportion of patients receiving OCSs decreased in every 6-month period post-mepolizumab initiation up to 24 months versus the 6 months pre-mepolizumab initiation (*P* = .014; Fig 1, *A*-*D*). There was also a significant decreasing trend in the mean daily dose of OCSs post-mepolizumab initiation versus the 6 months pre-mepolizumab initiation in both cohorts (*P* = .014). Significant decreases in both the proportion of patients receiving OCSs and the mean daily dose of OCSs were also observed in subgroups of patients with HES or EGPA who received a mepolizumab dose of 300 mg (see Table E1 in this article’s Online Repository at www.jaci-global.org). A significant reduction in the number of prescriptions for any nonsteroid immunosuppressant or cytotoxic treatments post-mepolizumab initiation was seen in patients with EGPA; this was not observed for the HES cohort (see Table E2 in this article’s Online Repository at www.jaci-global.org).

### Clinical outcomes

**Fig 2:**
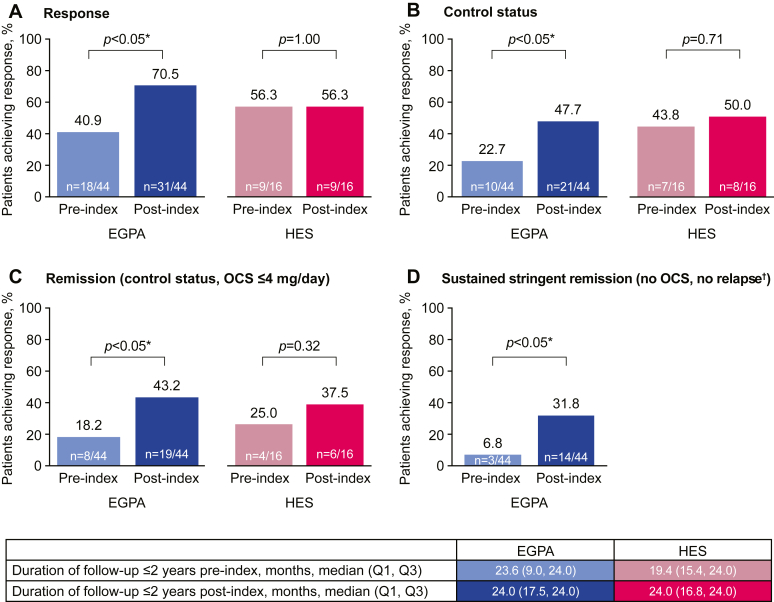
**A-D,** Response (*A*), control status (*B*), and remission outcomes (*C* and *D*) among patients with EGPA and HES within the 2-year period pre- and post-mepolizumab initiation. ∗*P* < .05. †In those with control status.

The proportion of patients with EGPA who achieved clinical response, increased control status, remission, and sustained stringent remission (OCS- and relapse-free) increased significantly within 2 years post- versus pre-mepolizumab initiation (Fig 2, *A-D*). Overall, a response was achieved by 18 (40.9%) patients with EGPA in the preindex period compared with 31 (70.5%) patients in the postindex period (*P* < .05). A total of 10 (22.7%) patients achieved control status preindex, whereas 21 (47.7%) patients attained control status postindex; the numbers achieving remission and sustained stringent remission increased significantly postindex. Statistically significant increases in all response and remission outcomes were also observed in the subgroup of patients with EGPA who received mepolizumab doses of 300 mg (see Table E3 in this article’s Online Repository at www.jaci-global.org). All 20 patients with EGPA who experienced remission pre- or post-mepolizumab initiation had BVAS-related clinical manifestations present within 4 weeks before remission and all symptoms were controlled at the time of remission (see Table E4 in this article’s Online Repository at www.jaci-global.org). Among patients with HES, there was no significant increase in the proportion of patients achieving response, control status, or remission (Fig 2, *A-C*), including in the 300-mg cohort (Table E3). Sustained stringent remission was evaluated only in the EGPA cohort because of sample size.

For patients with EGPA, during the preindex period, of the 10 patients who achieved control status, 40.0% experienced a relapse (required an OCS dose increase), whereas during the postindex period, of the 22 patients who achieved control status, 18.2% relapsed. Among patients with HES, 3 patients experienced flares preindex and 3 experienced flares postindex.

### End-organ manifestations

**Fig 3:**
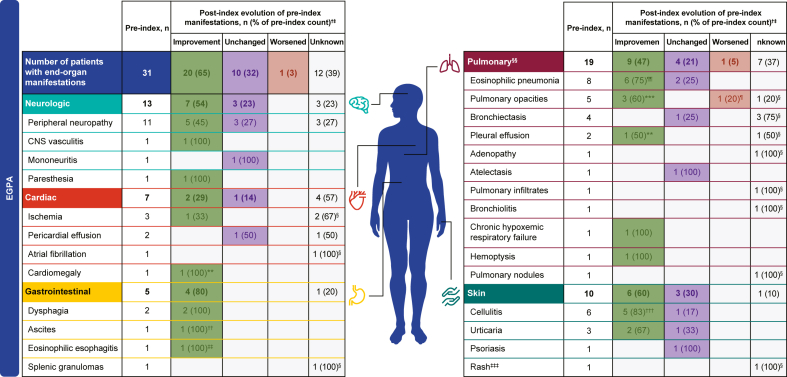
Evolution of end-organ damage manifestations post-mepolizumab initiation and objective measures of improvement in patients with EGPA.∗ *CNS*, Central nervous system; *CT*, computed tomography. ∗Patients could have multiple manifestations across different organs and within a specific organ. †The proportions of patients with improved, worsened, unchanged, or unknown manifestation status postindex were calculated using the preindex patient numbers as the denominator. The postindex evolvement columns evaluate only the status of manifestations present preindex in individual patients. ‡Other end-organ manifestations assessed for EGPA with n = 0 preindex and n = 1 postindex include carpal tunnel, premature ventricular contractions, and abdominal pain. §n ≥ 1 confirmed by objective measures postindex, but preindex status was unknown. ¶n = 1 confirmed by objective measures. ∗∗n = 1 confirmed by chest CT. ††n = 1 confirmed by abdominal CT. ‡‡n = 1 confirmed by esophageal biopsy. §§Of 19 patients reporting improvement on at least 1 pulmonary manifestation, 1 patient reported improved on pleural effusion and worsened on pulmonary opacities, and 1 patient reported unchanged on acute and chronic eosinophilic pneumonia and unknown on pulmonary nodules. ¶¶n = 4 confirmed by chest CT. ∗∗∗n = 2 confirmed by chest CT and n = 1 by both chest CT and chest x-ray. †††n = 2 confirmed by skin biopsy. ‡‡‡Rash was experienced by 2 patients with EGPA postindex.

Among patients with EGPA, most of the end-organ manifestations present in the preindex period were improved postindex, with some unchanged and only 1 that worsened (Fig 3). Overall, 31 (70.5%) patients experienced end-organ damage manifestations preindex compared with 11 (25.0%) postindex. In patients with EGPA treated with mepolizumab doses of 300 mg, similar values were observed. Neurologic, cardiac, gastrointestinal, pulmonary, and skin manifestations all decreased in the postindex period compared with the preindex period (Fig 3).

**Fig 4:**
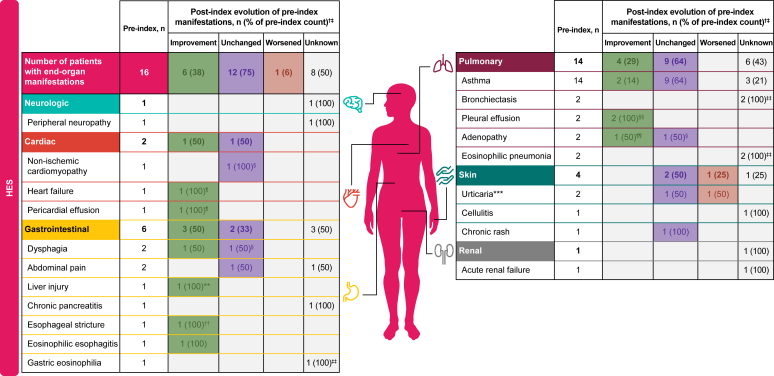
Evolution of end-organ damage manifestations post-mepolizumab initiation and objective measures of improvement in patients with HES.∗ *CNS*, Central nervous system; *CT*, computed tomography; *EGD*, esophagogastroduodenoscopy. ∗Patients could have multiple manifestations across different organs and within a specific organ. †The proportions of patients with improved, worsened, unchanged, or unknown manifestation status postindex were calculated using the preindex patient numbers as the denominator. The postindex evolvement columns evaluate only the status of manifestations present preindex in individual patients. ‡Other end-organ manifestations assessed for HES with n = 0 preindex and n = 1 postindex include angioedema. §n = 1 confirmed by objective measures. ¶n = 1 confirmed by echocardiogram. ∗∗n = 1 confirmed by serum transaminase elevation. ††n = 1 confirmed by EGD procedure. ‡‡n ≥ 1 confirmed by objective measures postindex, but preindex status was unknown. §§n = 1 confirmed by chest CT and n = 1 by chest x-ray. ¶¶n = 1 confirmed by chest CT. ∗∗∗Urticaria was experienced by 3 patients with HES postindex.

End-organ damage manifestations were more common in the preindex period for patients with HES, with 16 (100%) patients experiencing such manifestations compared with 13 (81.3%) patients in the postindex period, and improvements in a number of manifestations were confirmed by objective measures (Fig 4).

### Pulmonary function and asthma control

Of patients with EGPA and an asthma diagnosis, there were significant improvements in blood eosinophil count, pulmonary function, and asthma control scores post-mepolizumab initiation versus pre-mepolizumab initiation (see Table E5 in this article’s Online Repository at www.jaci-global.org). Median (Q1, Q3) blood eosinophil count decreased from 700.0 (366.0, 1530.0) cells/μL pre-mepolizumab to 100.0 (29.0, 202.3) cells/μL post-mepolizumab (*P* < .0001). Among patients with HES and an asthma diagnosis, there were numerical improvements in blood eosinophil count, pulmonary function, and asthma control scores post- versus pre-mepolizumab initiation; however, the 95% CIs were wide because of the small patient population (Table E5). Median (Q1, Q3) blood eosinophil count decreased from 1205.0 (228.0, 1660.0) cells/μL pre-mepolizumab to 125.0 (80.0, 791.0) cells/μL post-mepolizumab (*P* = .079).

### Patient improvements by outcomes (EGPA only)

**Fig 5:**
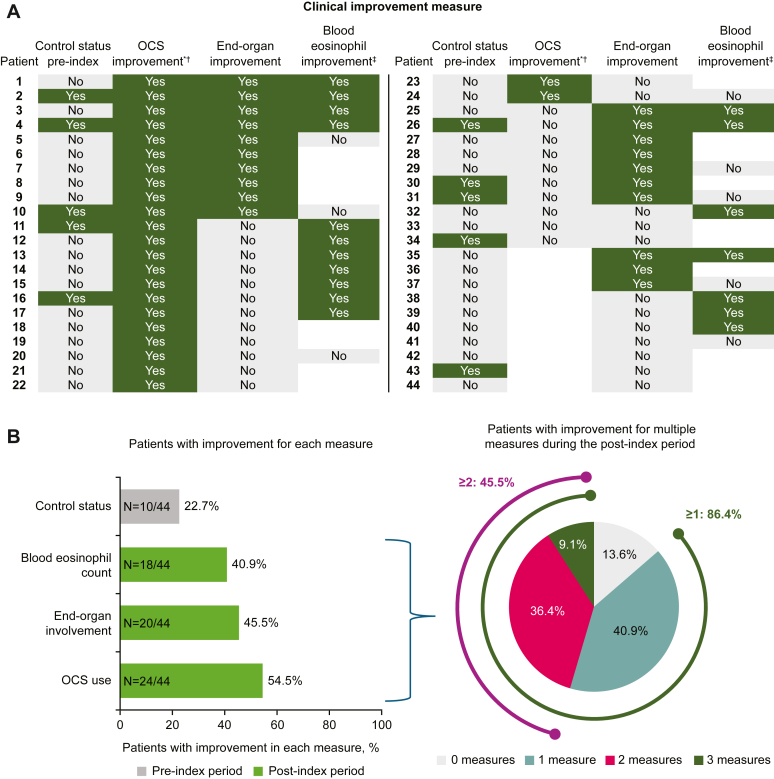
**A** and **B,** Individual (Fig 5, *A*) and summary (Fig 5, *B*) of patient improvements postindex in patients with EGPA. ∗Defined as OCS daily dose reduction within 12 months postindex vs daily dose within 12 months preindex. †Blank cells indicate no OCS use preindex. ‡Blank cells indicate missing values.

At the individual patient level, after mepolizumab initiation, 38 (86.4%) patients with EGPA had improvement in at least 1 of the outcomes of OCSs, end-organ manifestations, or blood eosinophil count (Fig 5, improvements shown in *green*). Of these, 4 (9.1%) patients had improvements in all 3 categories and a further 16 (36.4%) patients had improvements in 2 categories (Fig 5). The equivalent categorized data are not available for the HES cohort because of the small sample size.

## Discussion

Using clinical data from the largest US network of private allergy and immunology practices, these findings provide statistically significant evidence for the effectiveness of mepolizumab for EGPA in a real-world study and underscore the real-world benefits of mepolizumab in the management of EGPA or HES. Overall, this study provides evidence of benefit for reduction in OCS burden, measures of clinical response, and end-organ manifestations over 2 years of treatment, demonstrating that the benefits of mepolizumab for patients with EGPA or HES seen in large tertiary care centers are also seen in private practice settings.

The proportions of patients with EGPA or HES receiving OCSs both reduced significantly, and patients were able to decrease their OCS dose by 10 mg/d over 2 years, reducing cumulative exposure. Higher daily and cumulative OCS exposures are associated with a greater risk of adverse effects; therefore, expert consensus recommends minimizing maintenance doses.^8^^,^^23^ The OCS-sparing benefits of mepolizumab treatment for patients with EGPA have previously been demonstrated in clinical trials and real-world studies and to a more limited extent with HES.^12^^,^^19^^,^^20^^,^^24^, ^25^, ^26^, ^27^, ^28^ Although based on a small population, our data confirm the OCS-sparing effect of mepolizumab in EGPA and HES in real-world private practice settings.

In both the EGPA and HES cohorts, most of the patients remained on mepolizumab throughout the postindex period, suggesting that treatment was offering benefit. A mepolizumab dose of 300 mg administered subcutaneously was approved by the Food and Drug Administration for EGPA in December 2017 and for HES in September 2020,^13^^,^^14^^,^^29^^,^^30^ after phase 3 trials demonstrated efficacy versus placebo.^16^^,^^17^ Consequently, most patients received mepolizumab at the approved 300-mg dose for EGPA and HES; however, approximately one-third of patients received mepolizumab at the 100-mg dose approved for asthma and chronic rhinosinusitis with nasal polyps, with some later switching to mepolizumab 300 mg. There are several reasons why patients may have received the 100-mg dose: first, because the study period started in 2007, patients will have been initiated before approval of the 300-mg dose; second, treating physicians may have initially treated patients for asthma, until a diagnosis of EGPA was later confirmed, or chosen asthma dosing to focus treatment on the respiratory manifestations of EGPA; and third, there may have been payer restrictions on dosing.^31^^,^^32^ Mepolizumab was also available under a compassionate use program in the United States for patients with HES, with doses other than the approved 300-mg dose used before full approval from the Food and Drug Administration.^33^

Among patients with EGPA, significant improvements in clinical outcomes were observed in the 2 years after mepolizumab initiation compared with the 2 years before mepolizumab initiation. Strikingly, given the complex nature of this disease, 43% of patients achieved remission, with nearly a third of the patients achieving the goal of sustained stringent remission defined by control status, no OCS use, and no subsequent relapse. This treatment goal was based on expert opinion and aimed to capture the ambitious goal of eliminating OCS use. For patients with HES, there was a trend, without statistical significance, toward improvement in clinical outcomes (clinical response, control status, and remission). The lack of significance is likely attributable to the small number of patients with HES included in the study. This outcome may also be influenced by their lower use of immunosuppressants compared with patients with EGPA. Despite this, OCS-sparing benefit of mepolizumab was demonstrated in patients with HES, with significant reductions in OCS use in both the EGPA and HES cohorts.

Furthermore, almost two-thirds of patients with EGPA and more than a third of patients with HES had a documented improvement in end-organ manifestations. Mepolizumab treatment also significantly improved pulmonary function and asthma control in patients with EGPA, with numerical improvements shown for both outcomes in patients with HES. These results, suggesting benefits in symptom and disease control beyond medication-sparing outcomes, align with results from the MIRRA trial and other long-term and real-world studies of mepolizumab use in patients with EGPA.^17^^,^^20^^,^^34^, ^35^, ^36^, ^37^, ^38^

There are several limitations of this study that should be acknowledged. Data on medications prescribed outside of the Allergy Partners network may be incomplete, possibly affecting the results. The criteria used for this study to define HES did not include a requirement for biopsy-confirmed disease, which aligns with the International Cooperative Working Group definition of HES.^3^ This definition specifies the presence of blood and/or tissue hypereosinophilia with associated organ damage and exclusion of another underlying disorder or pathology as the primary driver of organ damage.^3^ Furthermore, in clinical practice, it is not always possible to perform biopsies because of factors such as risk to the patient or the need for urgent therapy, and biopsies may prove inconclusive.^3^ It should also be noted that recent phase 3 trials of biologics for HES have not required biopsy-confirmed disease for patient enrollment.^16^^,^^39^ The adjudication of clinical responses and control status is subject to evidence availability and subjective assessment by physicians; to mitigate this, expert clinicians served as centralized reviewers for all available data, providing a consistent basis for adjudication. Moreover, although the real-world study design is reflective of clinical practice, it may not account for all potential confounders. Nonetheless, in a pre-post study design, certain known or unknown confounders can be accounted for. In addition, sample sizes were small, particularly for patients with HES, and therefore results must be interpreted with care. However, the results may still be clinically relevant in this patient population.

These results further demonstrate the long-term OCS-sparing effects of mepolizumab in both EGPA and HES, additional clinical benefits beyond medication-sparing outcomes in patients with EGPA, and suggested clinical benefits with HES. Significant increases in response, control status, and remission were seen in the postindex period for the EGPA cohort. Moreover, most of the end-organ manifestations present in the preindex period improved in the postindex period in patients with EGPA, and manifestations were more common preindex than postindex for patients with HES. The data indicate that patients with rare eosinophilic diseases, such as EGPA and HES, are also managed by the wider allergy and immunology community of physicians in nonacademic private practices, rather than just at specialist academic centers. This emphasizes the broader need for allergists and immunologists to keep these rare diseases on their differential diagnoses.

**Clinical implications**

Mepolizumab treatment was associated with long-term OCS sparing and improvements in end-organ manifestations in patients with HES and EGPA managed in private practice, suggesting real-world clinical benefits and supporting previous findings.

## Disclosure statement

This study was funded by 10.13039/100004330GSK (GSK ID: 218960). The sponsor was involved in study design and implementation, as well as data collection, analysis, interpretation, writing the study report, and reviewing this article. The sponsor did not place any restrictions on access to data or statements made in the article. All authors had full access to the data on request and had final responsibility for the decision to submit for publication.

Disclosure of potential conflict of interest: M. E. Wechsler has received consulting and/or advisory honoraria from AbbVie, Allakos, Apogee, Areteia Therapeutics, Arrowhead Pharmaceutical, Avalo Therapeutics, Belenos Bio, Celldex, Connect Biopharma, Eli Lilly, Enveda Therapeutics, Equillium, General Medicines, Gilead, Jasper Therapeutics, Kinaset, Kymera, Merck, MyBiometry, Pfizer, Pharming, Phylaxis, Pulmatrix, Rapt Therapeutics, Recludix Pharma, Roche/Genentech, Sentien, Sound Biologics, Tetherex Pharmaceuticals, Uniquity Bio, Verona Pharma, and Zurabio; received consulting/advising/speaking honoraria from AstraZeneca, Amgen, Regeneron, GSK, and Sanofi/Genzyme and is doing research sponsored by them; has received stock options from Cellergy Pharma, Inc, and has received consulting honoraria, stock options and is doing research sponsored by Upstream Bio. A. Kovalszki has received funding from Blueprint Medicines and AstraZeneca; is part of an HES trial with AstraZeneca; is a consultant for GSK, ALK-Abelló, and the University of Michigan Inhale Collaborative Quality Initiative, sponsored by the Blue Cross Blue Shield of Michigan; and has received honoraria as the topic editor at DynaMed for Eosinophilia: Approach to the Patient. B. Stone is an employee of Allergy Partners, a consulting company that received payment from GSK to conduct this study (Allergy Partners is a registered trademark); and received consulting fees from Analysis Group. J. Silver was formerly employed by GSK and holds financial equities in GSK; and is now employed by Amgen and holds financial equities in Amgen. J. Hwee and A. G. Edgecomb are employed by GSK and hold financial equities in GSK. L. Huynh, W. da Costa Junior, S. Wang, and M. S. Duh are employees of Analysis Group, a consulting company that received payment from GSK to conduct this study; and have received research funds for previous studies from GSK, AbbVie, Apellis, AstraZeneca, Ayala Pharmaceuticals, Bayer, Blueprint Medicines, Humacyte, Janssen, Merck, Novartis, Pfizer, Sanofi, and Takeda. W. McCann is an employee of Allergy Partners; has received consulting fees from Aimmune, Regeneron, ARS Pharma, and AstraZeneca; and has received honoraria as a speaker for Amgen, AstraZeneca, ARS Pharma, and Regeneron. The rest of the authors declare that they have no relevant conflicts of interest.

Data-sharing statement: For requests for access to anonymized subject-level data, please contact the corresponding author.
