# Supplementary material for: Real-world outcomes and corticosteroid sparing with mepolizumab for EGPA or HES
Source: J Allergy Clin Immunol Glob. 2026 Mar 27;5(4):100695. doi: 10.1016/j.jacig.2026.100695 (PMC13141721; doi:10.1016/j.jacig.2026.100695)
Supplement: Supplementary Figs and Tables [file mmc1.docx]

**Real-world outcomes and corticosteroid sparing with mepolizumab for EGPA or HES**

**Online Repository**

**Contents**

**Table E1.** Trends in OCS use among patients with EGPA and HES treated with mepolizumab 300 mg from 6-months pre- and up to 24-months post-index 2

**Table E2.** Immunosuppressant treatment use pre- and post-index in EGPA and HES cohorts 3

**Table E3.** Response, control status and remission outcomes among patients with EGPA and HES within 2 years before and after mepolizumab 300 mg initiation 4

**Table E4.** BVAS clinical manifestations present within 4 weeks prior to remission date in patients with EGPA who experienced remission 5

**Table E5.** Changes in pulmonary function and asthma control among patients with asthma 6

**Figure E1.** Study design 7

**Figure E2.** Participant selection in the EGPA cohort 8

**Figure E3.** Participant selection in the HES cohort 9

**Plain language summary** 10

**Table E1.** Trends in OCS use among patients with EGPA and HES treated with mepolizumab 300 mg from 6-months pre- and up to 24-months post-index

|  | **Pre-index** | **Post-index** | | | | ***P*-value** |
| --- | --- | --- | --- | --- | --- | --- |
|  | **6-months** | **6-months** | **6–12 months** | **12–18 months** | **18–24 months** | - |
| **Patients with EGPA** | **n=30** | **n=33** | **n=30** | **n=25** | **n=21** | - |
| Patients with OCS use, n (%)* | 18 (60.0) | 16 (48.5) | 11 (36.7) | 5 (20.0) | 5 (23.8) | 0.050 |
| Daily OCS dose prednisone equivalent, mean (SD), mg/d | 15.5 (16.7) | 9.9 (11.2) | 8.8 (10.0) | 8.5 (10.1) | 6.4 (10.0) | 0.014 |
| **Patients with HES** | **n=12** | **n=11** | **n=11** | **n=9** | **n=7** | - |
| Patients with OCS use, n (%)^†^ | 9 (75.0) | 6 (54.5) | 4 (36.4) | 2 (22.2) | 1 (14.3) | 0.014 |
| Daily OCS dose prednisone equivalent, mean (SD), mg/d | 15.2 (14.9) | 7.9 (10.2) | 4.1 (5.6) | 3.7 (7.4) | 2.9 (7.6) | 0.014 |

*Among the 18 patients with EGPA who had OCS use in the 6-month pre-index period, 1 (5.6%) patient had no record of OCS use in the 24-month post-index period, and the patient reinitiated OCS 24 months after post-index initiation; ^†^Among the 9 patients who had OCS use in the 6-month pre-index period, 1 (11.1%) patient had no record of OCS use in the 24-month post-index period and had no record of OCS use after the 24-month post-index period.

EGPA, eosinophilic granulomatosis with polyangiitis; HES, hypereosinophilic syndrome; OCS, oral corticosteroid; SD, standard deviation.

**Table E2.** Immunosuppressant treatment use pre- and post-index in EGPA and HES cohorts

|  | **EGPA cohort**  **(N=44)** | | **HES cohort**  **(N=16)** | |
| --- | --- | --- | --- | --- |
|  | **Pre-index period*** | **Post-index period** | **Pre-index period*** | **Post-index period** |
| Number of immunosuppressant prescriptions, PPPY, mean (SD) | 0.4 (1.1) | 0.1 (0.4) | 0.1 (0.3) | 0.2 (0.7) |
| *P*-value | <0.05 | | 1.000 | |
| Patients with immunosuppressive/cytotoxic agent use, n (%) | 12 (27.3) | 7 (15.9) | 4 (25.0) | 2 (12.5) |
| Azathioprine | 8 (18.2) | 3 (6.8) | - | - |
| Methotrexate | 6 (13.6) | 5 (11.4) | 2 (12.5) | 0 |
| Mycophenolate | 3 (6.8) | 2 (4.5) | - | - |
| Cyclophosphamide | 3 (6.8) | 0 | - | - |
| Rituximab | 2 (4.5) | 1 (2.3) | - | - |
| Cyclosporine | 2 (4.5) | 0 | 0 | 1 (6.3) |
| Dapsone | 1 (2.3) | 0 | - | - |
| Leflunomide | 1 (2.3) | 1 (2.3) | - | - |
| Infliximab | 1 (2.3) | 0 | - | - |
| Hydroxyurea | - | - | 1 (6.3) | 1 (6.3) |
| Imatinib mesylate | - | - | 1 (6.3) | 1 (6.3) |

A dash signifies that no patients received the medication during either period. *Medication usage information was assessed any time before mepolizumab initiation based on structured data and chart review.

EGPA, eosinophilic granulomatosis with polyangiitis; HES, hypereosinophilic syndrome; PPPY, per person per year; SD, standard deviation.

**Table E3.** Response, control status and remission outcomes among patients with EGPA and HES within 2 years before and after mepolizumab 300 mg initiation

|  | **EGPA cohort**  **(N=34)** | | **HES cohort**  **(N=12)** | |
| --- | --- | --- | --- | --- |
|  | **Pre-index** | **Post-index** | **Pre-index** | **Post-index** |
| Response achieved, n (%) | 12 (35.3) | 24 (70.6) | 7 (58.3) | 8 (66.7) |
| *P*-value | <0.05 | | 0.705 | |
| Control status achieved, n (%) | 7 (20.6) | 16 (47.1) | 5 (41.7) | 7 (58.3) |
| *P*-value | <0.05 | | 0.414 | |
| Remission*, n (%) | 6 (17.6) | 14 (41.2) | 3 (25.0) | 5 (41.7) |
| *P*-value | <0.05 | | 0.157 | |
| Sustained stringent remission^†^, n (%)^‡^ | 2 (5.9) | 10 (29.4) | - | - |
| *P*-value | <0.05 | | - | |

*Control status achieved and prednisone-equivalent OCS dose ≤4 mg/d; ^†^Control status achieved, no OCS, and no relapse; ^‡^Evaluated only in the EGPA cohort due to sample size.

EGPA, eosinophilic granulomatosis with polyangiitis; HES, hypereosinophilic syndrome; OCS, oral corticosteroid.

**Table E4.** BVAS clinical manifestations present within 4 weeks prior to remission date in patients with EGPA who experienced remission*

| **Patients with BVAS clinical manifestations, n (%)** | **n=20** |
| --- | --- |
| **Chest** | 20 (100) |
| Wheezing | 20 (100) |
| Pulmonary infiltrate | 1 (5.0) |
| **Ear, nose and throat** | 12 (60.0) |
| Paranasal sinus involvement | 12 (60.0) |
| **Mucous membranes/eyes** | 5 (25.0) |
| Conjunctivitis/blepharitis/keratitis | 5 (25.0) |
| Blurred vision | 1 (5.0) |
| **Nervous system** | 4 (20.0) |
| Headache | 4 (20.0) |
| **Renal** | 2 (10.0) |
| Hypertension | 2 (10.0) |
| **General** | 1 (5.0) |
| Arthralgia/arthritis | 1 (5.0) |
| **Cutaneous** | 1 (5.0) |
| Other skin vasculitis | 1 (5.0) |

*Pre- or post-mepolizumab initiation, 20 patients had remission, and all had BVAS clinical manifestations present within 4 weeks prior to remission.

BVAS, Birmingham Vasculitis Activity Score; EGPA, eosinophilic granulomatosis with polyangiitis.

**Table E5.** Changes in pulmonary function and asthma control among patients with asthma

|  | **EGPA cohort**  **(N=43)** | **HES cohort**  **(N=14)** |
| --- | --- | --- |
| Blood eosinophil count, cells/µL |  |  |
| Pre-index period*, n (%) | 39 (90.7) | 14 (100) |
| Median (Q1, Q3) | 700.0 (366.0, 1530.0) | 1205.0 (228.0, 1660.0) |
| Post-index period | 28 (65.1) | 12 (85.7) |
| Median (Q1, Q3) | 100.0 (29.0, 202.3) | 125.0 (80.0, 791.0) |
| *P*-value | <0.0001 | 0.079 |
| Asthma control test scores, n (%) |  |  |
| Pre-index period | 23 (53.5) | 7 (50.0) |
| Median (Q1, Q3) | 17.0 (11.0, 24.0) | 22.0 (20.0, 25.0) |
| Post-index period | 38 (88.4) | 12 (85.7) |
| Median (Q1, Q3) | 24.0 (22.0, 24.0) | 24.5 (22.5, 25.0) |
| *P*-value | <0.001 | 0.063 |
| FEV_1_ % predicted, n (%) |  |  |
| Pre-index period | 34 (79.1) | 12 (85.7) |
| Median (Q1, Q3) | 70.2 (50.2, 77.2) | 77.6 (72.1, 85.8) |
| Post-index period | 32 (74.4) | 11 (78.6) |
| Median (Q1, Q3) | 81.5 (74.3, 95.7) | 86.8 (75.6, 91.1) |
| *P*-value | <0.0001 | 0.301 |

*The closest laboratory assessment to the index date was reported during any time prior to the index date.

EGPA, eosinophilic granulomatosis with polyangiitis; FEV_1,_ forced expiratory volume in 1 second; HES, hypereosinophilic syndrome; Q, quartile.

**Figure E1.** Study design


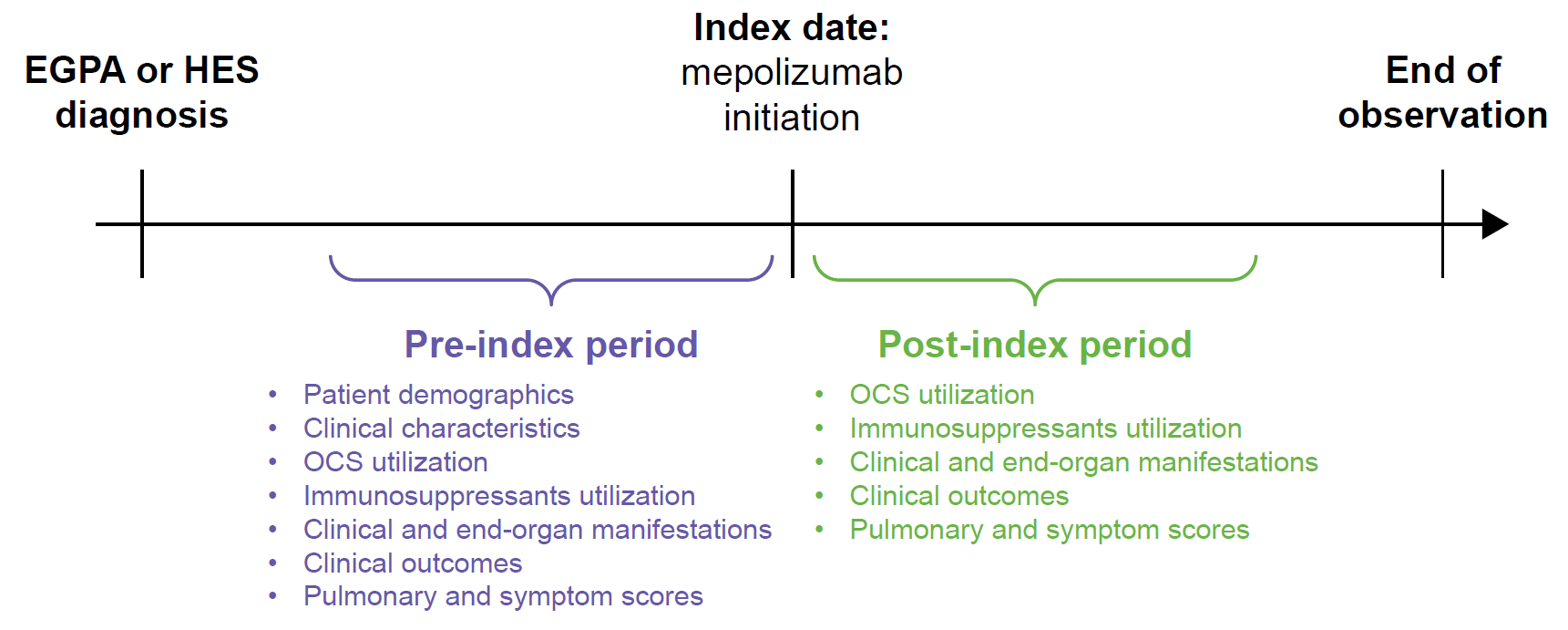


EGPA, eosinophilic granulomatosis with polyangiitis; HES, hypereosinophilic syndrome; OCS, oral corticosteroid.

**Figure E2.** Participant selection in the EGPA cohort


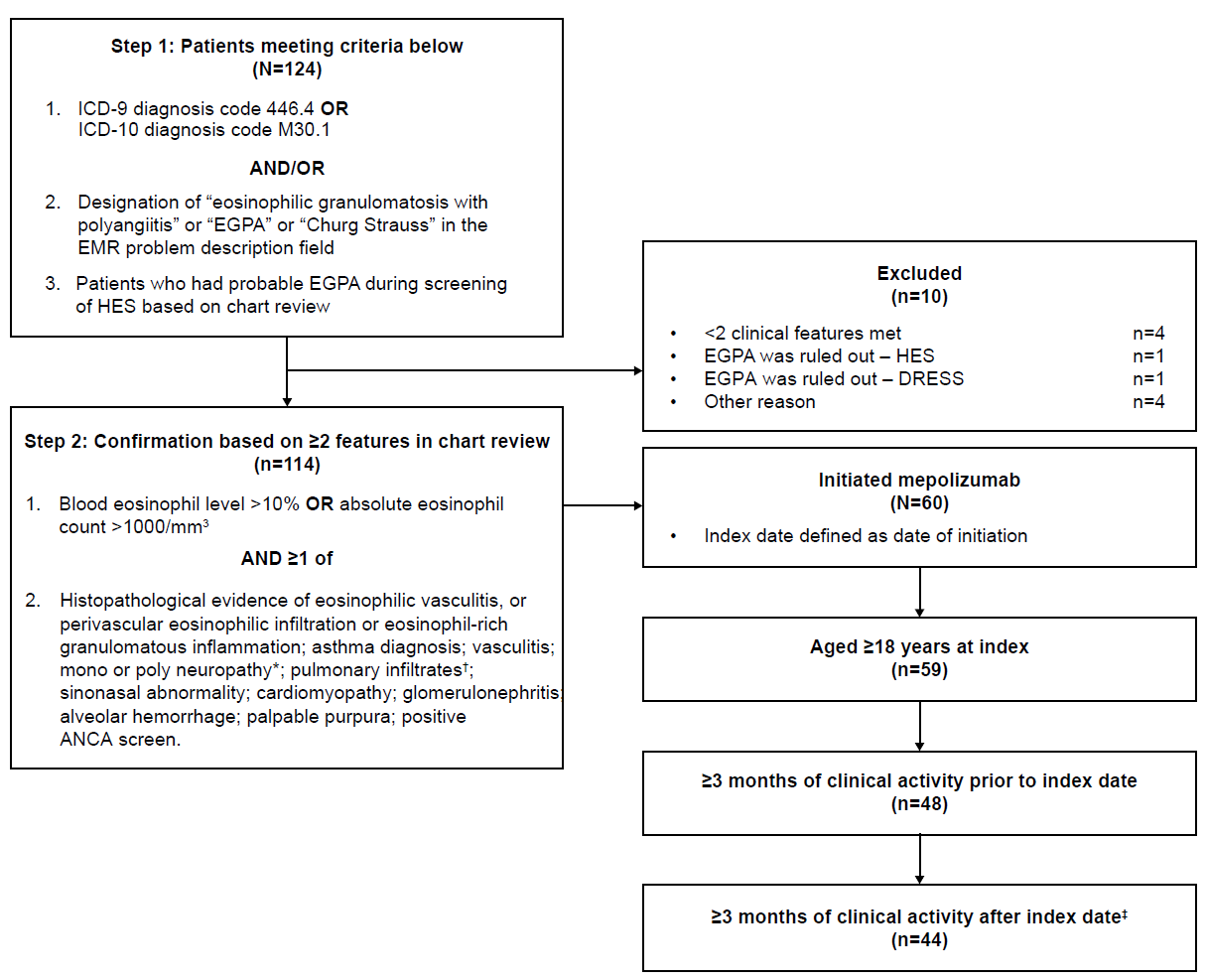


*Motor deficit or nerve conduction abnormality; ^†^Nonfixed; ^‡^Unless patient died.

ANCA, anti-neutrophil cytoplasmic antibody; DRESS, drug reaction with eosinophilia and systemic symptoms; EGPA, eosinophilic granulomatosis with polyangiitis; EMR, electronic medical record; HES, hypereosinophilic syndrome; ICD, International Classification of Diseases.

**Figure E3.** Participant selection in the HES cohort


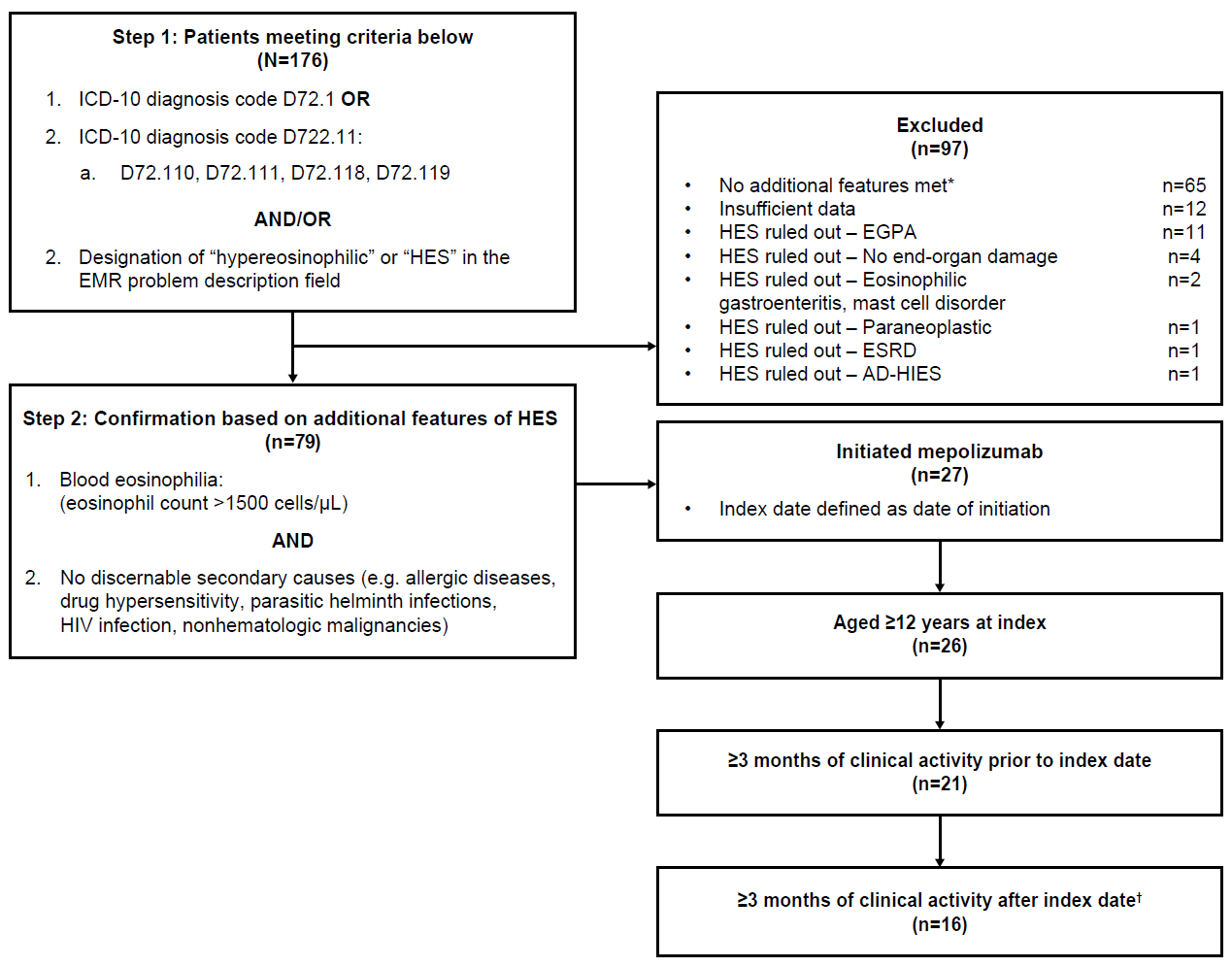


*Included patients who did not have any documented laboratory results with eosinophil count ≥1500 cells/μL, eosinophil count elevation was not persistent, and eosinophil count related to asthma, severe allergic disease or drug reaction; ^†^Unless patient died.

AD-HIES, autosomal dominant hyper IgE syndrome; EGPA, eosinophilic granulomatosis with polyangiitis; EMR, electronic medical report; ESRD, end-stage renal disease; HES, hypereosinophilic syndrome; ICD, International Classification of Diseases; IgE, immunoglobulin E.

## **Plain language summary**

Eosinophilic granulomatosis with polyangiitis (EGPA) and hypereosinophilic syndrome (HES) are rare conditions in which too many eosinophils (a type of white blood cell) can damage organs. Mepolizumab is a targeted medicine (a biologic) that lowers eosinophil levels. Steroids are also used to treat HES and EGPA but can cause side effects, especially with long-term use.

We looked at medical records from a large US allergy practice to see what happened after people with EGPA or HES started mepolizumab as part of their normal treatment, compared with before treatment. This study was funded by GSK.

The study included 16 people with HES and 44 people with EGPA. After starting mepolizumab, fewer people with HES or EGPA needed steroids and the average daily steroid dose fell over the following two years. More than twice as many people with EGPA had controlled or improved symptoms and more achieved remission after starting mepolizumab treatment. Signs of organ involvement also fell from 7 in 10 people with EGPA before starting mepolizumab to 1 in 4 people after treatment. This change included improvements in symptoms affecting the lung, heart, skin, gut and the nervous system. For people with HES some improvements in signs of organ involvement were seen, but numbers were smaller.

This study showed that patients taking mepolizumab as part of routine treatment were able to reduce steroid use and had improvements in symptoms.
